# Refractory topiramate-induced angle-closure glaucoma in a man: a case report

**DOI:** 10.1186/1752-1947-5-33

**Published:** 2011-01-26

**Authors:** Matthew C Willett, Deepak P Edward

**Affiliations:** 1 Summa Health Systems, 75 Arch St. Suite 512, Akron, OH 44304, USA

## Abstract

**Introduction:**

Topiramate is a sulphonamide derivative indicated in the treatment of epilepsy and migraine. A known adverse affect is an idiosyncratic reaction that results in angle-closure glaucoma. We describe a patient with bilateral glaucoma related to topiramate that showed some unusual clinical features.

**Case presentation:**

A 39-year-old Caucasian man presented with acute angle-closure glaucoma; he initially presented with intractable headaches after being treated with an escalating dose of topiramate. Clinical signs included elevated intraocular pressure that was initially refractory to treatment, shallow anterior chambers, and extensive bilateral choroidal effusions. After treatment with intravenous methylprednisolone, in conjunction with conventional glaucoma treatment, there was rapid reduction of intraocular pressure, gradual delayed resolution of the choroidal effusion and induced myopic shift; and eventually a good outcome without optic nerve damage.

**Conclusion:**

This case illustrates the importance of recognizing this entity in a non-ophthalmic setting and that intravenous methylprednisolone may be useful in the treatment of the condition when it is not responsive to conventional treatment. In addition, it is important to recognize that complete resolution of visual symptoms from the myopic shift may be delayed, despite normalization of intraocular pressure.

## Introduction

We report a case of topiramate-induced angle-closure glaucoma (TiACG) that was initially refractory to conventional treatment. This case illustrates the importance and method of timely and proper diagnosis, as well as management of such cases that might initially respond poorly to treatment.

## Case presentation

A 39-year-old Caucasian man, with a past medical history of hypertension and migraine headaches, presented to the emergency department with a one-day history of left-sided headache with blurred vision and haloes around lights, which rapidly progressed to involve the right side. After initial computed tomography of the head, which was unremarkable, he was admitted to the hospital with the diagnosis of intractable headaches and treated with intravenous morphine and topiramate. The following morning, the patient's ocular complaints worsened, and the ophthalmology service was consulted.

**Figure 1:**
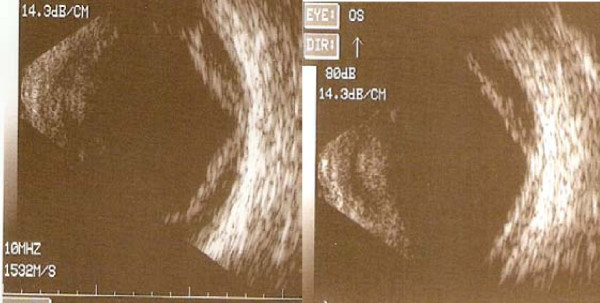
**B-scan ultrasound of the left eye showing moderately elevated choroidal effusions that extend into the posterior pole**.

Further questioning revealed that the patient had been started on topiramate, 25 mg, one week before presentation for migraine headaches, and had been instructed to double the dose one day before the onset of symptoms. On initial examination, the best-corrected visual acuity (BCVA) was 20/100 in both eyes. The intraocular pressures (IOPs) by tonopen were 70 mm Hg in both eyes. Slit-lamp examination revealed bilateral moderate conjunctival injection, moderate to severe corneal edema, and fixed mid-dilated pupils. The anterior chambers in both eyes were shallow but without iridocorneal touch or discernable inflammation. Gonioscopy with and without compression revealed appositional angles. A limited view to the posterior pole was available. B-scan ultrasound showed moderate serous choroidal effusions in both eyes that extended to the posterior pole (Figure 1). The patient was diagnosed with angle-closure glaucoma, likely secondary to topiramate treatment.

After discontinuation of the topiramate, the patient was treated with topical timolol maleate, 0.5%, dorzolamide hydrochloride, 2%, brimonidine tartrate, 0.15%, homatropine, 2%, prednisolone acetate, 1%, and 500 mg acetazolamide orally. After one hour, the topical treatment was repeated with the same combination, and no change in IOP was noted. Intravenous 20% mannitol, 100 g in 500 ml, was administered, and three hours later, IOP was 51 mmHg in the right eye and 83 mmHg in the left eye. Given the continued IOP elevation and the severity of the choroidal effusions, the patient was given methylprednisolone, 250 mg IVPB, and the previously outlined regimen was continued. By the next morning the patient's pain score was 0/10 in the right eye and 4/10 in the left eye. The BCVA at that visit was 20/60, right eye, and counting fingers, left eye. Retinoscopy of the right eye was -5.00 D, consistent with anterior displacement of the crystalline lens, and was unsuccessful in the left eye secondary to corneal edema. The patient was previously emmetropic. At that visit, the IOP values were 38 mm Hg, right eye, and 37 mm Hg in the left eye by pneumotonometer. Anterior-segment examination showed persistent bilateral shallowing of the anterior chamber with microcystic epithelial edema and bullae bilaterally with descemet folds in the left eye only. The patient was given a second dose of methylprednisolone, 250 mg IVPB, and discharged home with a regimen of atropine 1%, Cosopt (2% dorzolamide; 0.5% timolol; Merck Inc, NJ), bimatoprost, 0.03%, and prednisolone acetate, 1% drops. The patient was followed up daily with gradual reduction of intraocular pressures. At one week after the initial presentation, the patient's best corrected visual acuity was 20/15 in the right eye and 20/20 in the left eye with -4.00 D sphere both eyes. The intraocular pressures were 12 mm Hg, right eye, and 16 mm Hg, left eye, and gonioscopy showed open angles bilaterally. The anterior-chamber depth improved, and choroidal effusions were decreased on fundoscopic examination, eventually resolving by the two-week visit. The myopic shift continued to decrease, and by one month, the patient's uncorrected visual acuity had returned to 20/15 in both eyes, and intraocular pressures without hypotensive medications were 10 mm Hg right eye and 11 mm Hg left eye. Anterior examination was significant for glaucomflecken bilaterally and localized posterior synechiae in the left eye. Dilated fundus examination showed that the cup-to-disk ratio was 0.2 with intact rim and a full visual field in both eyes.

## Discussion

We report on a case of severe bilateral TiACG with extensive choroidal effusions. Fraunfelder *et al. *[1] reviewed 115 reports of TiACG and found that 85% of cases occurred within the first two weeks of treatment. This is consistent with the presentation in this patient, who had been on treatment for one week and experienced the onset of symptoms within one day of doubling his dose. Previous reports have not identified a minimal dose of topiramate associated with ACG, and the Fraunfelder *et al. *study showed that almost 50% of cases occur with doses of 50 mg or less. It is possible that the angle-closure glaucoma in our patients may have been precipitated when the dose was doubled [1].

The principal step in appropriate management of TiACG is making a correct diagnosis. We suggest that an earlier diagnosis and treatment at the time of the patient's presentation to the emergency department might have allowed faster resolution of the episode of angle closure. It is recommended that a patient with angle-closure glaucoma, especially if it is bilateral, should have a detailed history and review of the medication list for topiramate or other sulfonamide derivatives that cause such idiosyncratic uveal effusions. Refraction and B-scan ultrasonography should then be performed, looking for myopic shift and ciliochoriodal effusion, which help differentiate TiACG from typical pupillary block ACG [2].

An interesting feature in our patient was the large and extensive posterior choroidal effusion that was demonstrated in both eyes. In TiACG, the uveal effusions reported are typically anterior, causing shallowing of the anterior chamber [1]. Our patient presumably also had bilateral anterior uveal effusions, accounting for the anterior chamber shallowing seen clinically. However, ultrasound biomicroscopy was not performed in our patient. Posterior uveal effusions have been reported in TiACG but are often shallow in nature. Large choroidal effusions from other causes are often associated with lower IOP, which may be associated with aqueous hypersecretion or ciliary body detachment [3]. It is unclear whether the choroidal effusions in TiACG are associated with normal aqueous humor production or hyposecretion. It is, however, likely that mechanical angle closure by anterior displacement of the iris lens diaphragm may override any other factors that affect aqueous dynamics, resulting in IOP elevation.

The anterior displacement of the iris lens diaphragm caused a myopic shift in the patient's refraction and, as illustrated by the temporal course in this patient, the myopic shift may take several weeks to resolve, despite normalization of IOP. This might be a useful observation to discuss with the patient during the recovery phase.

The treatment of TiACG is tailored to the severity of IOP elevation. The first step is immediate discontinuation of topiramate. This step combined with topical hypotensive medications and cycloplegics is most often sufficient to reduce the IOP. In a large retrospective case series, the typical course of IOP normalization in TiACG was reported to be within hours to days after cessation of topiramate and initiating of conventional IOP-reducing therapy [1]. In contrast, our case of bilateral TiACG necessitated further treatment to correct the marked elevation of IOP that appeared to be refractory to conventional therapy over many hours. Rhee and colleagues [4] suggested that in cases that do not respond to conventional therapy, systemic corticosteroids and hyperosmolar agents should be used to speed recovery and to avoid the need for surgical intervention. This approach was used in our patient, and recovery followed after administration of two doses of methylprednisolone. Although one could argue that the cessation of topiramate resulted in the resolution of the symptoms and signs, given the severity of the presentation in this case and the low side-effect profile associated with the pulse doses of methylprednisolone, it is reasonable to consider this as a treatment option.

## Conclusion

The use of topiramate may lead to a spectrum of ocular symptoms ranging from myopic shift to secondary angle-closure glaucoma, secondary to ciliochoroidal effusions. In cases of severe disease refractory to conventional treatment, alternative options could include the use of hyperosmotics and possibly intravenous methylprednisolone to speed recovery.

## Consent

Written informed consent was obtained from the patients for publication of this case and any accompanying images. A copy of the written consent is available for review by the Editor-in-Chief of this journal.

## Competing interests

Neither author has any conflicting interests.

## Authors' contributions

Both authors contributed equally to the manuscript. Both authors read and approved the final manuscript.
